# Associations Between Fluctuations in Daytime Sleepiness and Motor and Non‐Motor Symptoms in Parkinson's Disease

**DOI:** 10.1002/mdc3.13102

**Published:** 2020-10-26

**Authors:** Arja Höglund, Peter Hagell, Jan‐Erik Broman, Sven Pålhagen, Kimmo Sorjonen, Sten Fredrikson, Per Svenningsson

**Affiliations:** ^1^ Department of Clinical Neuroscience Karolinska Institutet Stockholm Sweden; ^2^ Department of Neurology Karolinska University Hospital Huddinge Stockholm Sweden; ^3^ The PRO‐CARE Group, Faculty of Health Sciences Kristianstad University Kristianstad Sweden; ^4^ Department of Neuroscience, Psychiatry Uppsala University Uppsala Sweden

**Keywords:** daytime sleepiness, Parkinson's disease, motor fluctuations and non‐motor fluctuations, home diary, PKG

## Abstract

**Background:**

Non‐motor fluctuations are a major concern in Parkinson's disease (PD), and they have been categorized into neuropsychiatric, autonomic and sensory fluctuations. However, this categorization does not include sleep and sleep‐related features, and the association between daytime sleepiness and other motor and/or non‐motor fluctuations in PD remains to be elucidated.

**Objective:**

To investigate the relationship between daytime sleepiness and other non‐motor and motor fluctuations in people with PD.

**Methods:**

A three‐day home diary recording daytime sleepiness, mood, anxiety, and motor symptoms was used along with the Karolinska Sleepiness Scale (KSS) and 6 days of accelerometer (Parkinson's KinetiGraph™; PKG™) registration to detect motor fluctuations among people with a DaTSCAN verified clinical PD diagnosis (32 men; mean PD duration, 8.2 years). Participants were categorized as motor fluctuators or non‐fluctuators according to the UPDRS part IV and/or the presence of motor and non‐motor fluctuations.

**Results:**

Fifty‐two people with PD participated. Daytime sleepiness correlated significantly with motor symptoms, mood and anxiety among those classified as motor fluctuators (n = 28). Motor fluctuators showed stronger correlations between the individual mean level of all diary variables (daytime sleepiness, anxiety, mood and motor symptoms) when compared to the non‐fluctuators (n = 24). Stronger positive within‐individual correlations were found among fluctuators in comparison to non‐fluctuators. In general, PKG data did not correlate with diary data.

**Conclusion:**

Episodes of daytime sleepiness, as reported by home diaries, were associated with other self‐reported non‐motor and motor fluctuations, but were not supported by PKG data.

Parkinson's disease (PD) is diagnosed based on the presence of bradykinesia, rigidity or tremor and with non‐motor symptoms (NMS) included to support the diagnosis.^1^, ^2^ NMS include cognitive decline, sleep problems, dysautonomia and neuropsychiatric symptoms.^3^ It is a well‐known phenomenon that motor symptoms fluctuate in a large proportion of patients with PD and this depends on the natural progression of the disease as well as the response to treatment.^4^ During recent decades, there has been growing interest in non‐motor fluctuations (NMF) in PD. These have been separated into three categories: neuropsychiatric, autonomic and sensory fluctuations.^5^

There are only few prospective studies on NMF in PD. Richard et al.^6^ studied mood and anxiety along with motor fluctuations in PD using a home diary for seven consecutive days. Fluctuations occurred in 35% of people with PD. Anxiety (29%) was the most common fluctuating symptom followed by motor (24%) and mood (21%) fluctuations; about a third of fluctuators reported fluctuations of all three symptoms.^6^ Ossig and colleagues^7^ used a 5‐day home diary to detect the temporal connection between motor and NMS (neuropsychiatric, autonomic and sensory) fluctuations in people with advanced PD. They found no evidence of NMF without motor fluctuations, but found that NMF occurred largely independently of motor fluctuations.

Sleep disturbances and daytime sleepiness have not been included in any NMF category because sleep patterns can be a consequence of other parkinsonian symptoms, such as nocturnal akinesia, and can be improved by treatment with dopaminergic medication.^4^ However, daytime sleepiness may have an association with external stimuli other than dopamine‐related fluctuations, including dysfunctional circadian rhythmicity.^8^ It is therefore important to evaluate whether daytime sleepiness is associated with other motor and/or non‐motor fluctuations in PD. In the present study we used a home diary to evaluate daytime sleepiness, other non‐motor (mood and anxiety) and motor fluctuations, as well as a wearable accelerometer to investigate the relations between variations in daytime sleepiness, and non‐motor and motor fluctuations.

## Methods

### Participants

All participants were recruited from a movement disorders outpatient unit in Stockholm, Sweden. The inclusion criterion for this study was a DaTSCAN verified clinical PD diagnosis.^9^ Exclusion criteria were a documented diagnosis of dementia, severe untreated depression, and inability to understand the Swedish language. Sixty‐eight people with PD, who met the inclusion and exclusion criteria, were invited to participate in the study by a mailed letter. Seven did not respond to the invitation, and eight did not consent. Fifty‐three people were included in the study from September 2015 to October 2016.

### Instruments

At an outpatient visit, participants completed questionnaires regarding daytime sleepiness (Epworth Sleepiness Scale; ESS,^10^), sleep quality (the Pittsburgh Sleep Quality Index, PSQI,^11^); REM sleep behavior disorders single questionnaire (RBD1Q^12^;), fatigue (the Functional Assessment of Chronic Illness Therapy – Fatigue scale, FACIT‐F,^13^), and anxiety and depression symptoms (The Hospital Anxiety and Depression Scale, HADS,^14^). For all instruments, except the FACIT‐F, higher scores reflect more pronounced symptoms. Cognitive status was assessed using the Mini Mental State Exam (MMSE; higher scores = better).^15^

We constructed a self‐reported home diary for daytime registration of motor and non‐motor symptoms during three consecutive days, of which one was either a Saturday or a Sunday. Motor symptoms were defined as bradykinesia, tremor, and rigidity, and non‐motor symptoms were divided into feelings of (1) worrying, nervousness and anxiety; (2) low mood; and (3) sleepiness. The occurrence of these symptoms was rated every second hour from awakening to bed time, as “not at all”, “somewhat”, “pretty much” or “very much”. In addition, the diary included the Karolinska Sleepiness Scale (KSS) where participants rate their levels of sleepiness from 1 (very alert) to 9 (extremely sleepy), which makes it possible to capture variations in sleepiness over the day.^16^

Motor symptoms were also recorded using the Parkinson's KinetiGraph (PKG™; Global Kinetic Corporation), which provides dyskinesia (DKS) and bradykinesia (BKS) scores.^17^ Variations of DKS and BKS can be used to calculate a fluctuation score (FDS); an FDS ≥7.8 has been suggested to represent the presence of fluctuations.^18^ The PKG‐derived proportion of time as immobile (PTI; defined as ≥2 minutes of immobility) has been suggested as an indicator of daytime sleepiness or somnolence.^19^ The PKG report is a summary of average scores from data collected over 6 days during the daytime from 0900 to 1800. Results are presented as a summary score for BKS, DKS and FDS, and as the percentage of time immobile for PTI.Parkinsonian motor symptoms were assessed using part III (motor exam) of the Unified PD Rating Scale (UPDRS^20^). Hoehn and Yahr staging^21^ was used as an indicator of PD severity. The presence of motor fluctuations was defined according to the UPDRS part IV: items 36, (predictable “off”), 37 (unpredictable “off”), 38 (sudden “off”) and 39 (waking day “off”‐time); those scoring ≥1 were considered fluctuators. We also asked about the overall presence of motor and non‐motor fluctuations, because UPDRS covers the past week and it is well‐known that a people with PD can under‐ and over‐estimate their symptoms.^22^

### Procedure

During the inclusion visit all participants were clinically assessed according to the UPDRS, Hoehn and Yahr and MMSE during the “on” phase. All medications and co‐morbidities were recorded. Measurements of orthostatic blood pressure and weight were made. Following training on how to complete the home diary and handle the PKG device, these were provided to the participants and the PKG device was activated. All assessments and training were performed by the same experienced specialized PD nurse (A.H.). In parallel to the home diary, the participant carried a PKG device on their most affected upper limb for 6 days, including the three consecutive home diary days. Participants who were treated with oral levodopa (n = 41) had the dosage times pre‐programmed on their PKG device. After the study period the PKG and the diary were returned by mail.

### Statistics

Data describing frequencies, means, standard deviations and comparisons between fluctuators, and non‐fluctuators were conducted using Chi‐square, Mann‐Whitney U‐ and t‐tests, as appropriate. Anti‐parkinsonian medications were expressed as total daily levodopa equivalent doses (LED),^23^ as well as for levodopa and dopamine agonists separately.

For each participant we calculated the within‐individual mean (WIM) and the within‐individual standard deviation (WISD), as well as the within‐individual correlation Spearman's r, (WIRS) between all diary variables. WIM indicates the participant's average level of self‐rated symptoms while WISD is a measure of how much these ratings fluctuate within each participant during the study period. WIRS indicates how each participant's ratings tend to covary, with a positive value showing that a high rating on one variable tends to coincide with a high rating on the other variable, and a negative value indicating that a high value on one variable tends to coincide with a low value on the other variable.

Analyses were conducted using IBM SPSS Statistics 26, and R 3.5.0 statistical software,^24^ employing the weights^25^ and beanplot^26^ packages. The alpha level of significance was set at 0.05 (2‐tailed). Since this is an exploratory study, we did not correct *P*‐values for multiple comparisons.

## Results

Forty‐nine people completed the home diary for three (n = 42), 2–2.5 (n = 5) and 0.5–1.5 (n = 2) days, and 52 PKG registrations were completed and included in the analysis. Thus, three people underwent PKG registration but did not complete the diary. One people did not complete either the home diary or the PKG and was excluded.

Participant characteristics are summarized in Table 1. Twenty‐eight participants were classified as motor fluctuators and 24 as non‐fluctuators. Motor fluctuators had longer PD duration, more advanced PD, more sleep‐related problems, and fatigue, and were treated with higher dosages of levodopa. However, they reported less anxiety compared to non‐fluctuators.

**TABLE 1:** *Characteristics for all participants (n = 52) divided into fluctuators (n = 28) and with non‐fluctuators (n = 24)*

Variable	All participants (n = 52)	Fluctuators (n = 28)	Non‐fluctuators (n = 24)	*P*‐value
Gender, male, n (%)	32 (61.5%)	15 (53.6%)	17 (70.8%)	0.202*
Age (years), mean (SD)	65.3 (10.5)	63.8 (11.6)	67.0 (8.9)	0.260^#^
Time since PD diagnosis (years), mean (SD)	8.2 (7.7)	11.9 (8.2)	4.0 (4.3)	<0.001^#^
Daily LDE dose (total; mg), mean (SD)^b^	816.6 (586.2)	1122.4 (627.0)	459.8 (231.9)	<0.001^#^
Daily LDE dose (levodopa; mg), mean (SD)^c^	615.8 (571.2)	879.2 (647.8)	308.2 (218.2)	<0.001^#^
Daily LDE dose (dopamine agonists; mg), mean (SD)^d^	103.6 (128.4)	73.2 (118.2)	139.1 (133.1)	0.067^#^
Orthostatic hypotension, n (%)^e^	13 (25%)	10 (35.7%)	3 (12%)	0.054*
Mini Mental State Examination (0–30)^f^ ^,^ ^i^	28.3 (19–30)	27.9 (19–30)	28.7 (26–30)	0.285^§^
Body Mass Index, mean (SD)	24.1 (4.1)	23.2 (4.1)	25.2 (3.9)	0.075^#^
Hoehn & Yahr stage of PD in ON (I–V)^f^ ^,^ ^g^ ^,^ ^h^	II (I‐IV)	III (I‐IV)	II (I‐III)	0.018^§^
Hoehn & Yahr stage of PD in OFF (I–V)^g^ ^,^ ^h^	III (III‐V)	III (III‐V)	NA	NA
UPDRS III, motor score (0–108)^f^ ^,^ ^h^	20.3 (2–49)	22.5 (2–49)	17.7 (4–37)	0.054^§^
UPDRS IV, dyskinesia score (0–13)^h^	1.2 (0–6)	1.6 (0–6)	0.6 (0–2)	0.003^§^
UPDRS IV, motor fluctuation score (0–7)^h^	1.5 (0–4)	2.6 (2–4)	0.2 (0–2)	<0.001^§^
ESS daytime sleepiness score (0–24)^h^	10.1 (0–23)	10.3 (0–20)	9.9 (0–23)	0.451^§^
EDS (ESS score >10), n (%)	22 (42.3%)	16 (57.1%)	6 (24%)	0.019*
PSQI, sleep quality score (0–21)^h^	10.5 (6–17)	11.4 (9–17)	9.4 (6–14)	0.001^§^
RBDQ1, REM sleep behavior disorder, n (%)	19 (36.5%)	12 (42.9%)	7 (29.2%)	0.307*
FACIT‐F, fatigue score (0–52)^i^	36.0 (12–52)	32.2 (11.9)	40.4 (7.2)	0.010^§^
HADS, depression score (0–21)^h^	9.1 (6–15)	9.0 (8–13)	9.1 (7–15)	0.507^§^
HADS, anxiety score (0–21)^h^	13.0 (8–17)	12.4 (8–16)	13.8 (9–17)	0.040^§^
PKG, bradykinesia score (BKS)^a^	27.1 (2.3)	24.6 (7.6)	30.0 (6.0)	0.002^§^
PKG dyskinesia score (DKS)^a^	4.0 (6.0)	5.6 (7.6)	2.1 (2.0)	0.042^§^
PKG fluctuations score (FDS)^a^	9.3 (4.1)	10.8 (4.7)	7.4 (2.1)	0.003^§^
PKG proportion of time immobile (PTI) (%)^a^	6.0 (5.0%)	4.9 (4.1%)	7.1 (5.6%)	0.108^§^

^a^ Data are mean (standard deviation; SD) unless otherwise noted.

^b^ Including all antiparkinsonian medications; derived according to Tomlinson et al.^23^.

^c^ Including only levodopa (and associated enzyme inhibitors); derived according to Tomlinson et al.^23^.

^d^ Including only dopamine agonists; derived according to Tomlinson et al.^23^.

^e^ Orthostatic hypotension: a systolic blood pressure decreases of at least 20 mm Hg or a diastolic blood pressure decrease of at least 10 mg Hg within 3 minutes of standing according the American Academy of Neurology.^33^.

^f^ As assessed during the “on” phase.

^g^ Range, I–V (I, Mild unilateral disease; II, Bilateral disease without postural impairment; III, Bilateral disease with postural impairment, moderate disability; IV, Severe disability, still able to walk and stand unassisted; V, Confined to bed or wheelchair unless aided).

^h^ High scores = more problems.

^i^ Higher scores = less problems.

* Chi‐square test.

^#^ Unpaired two‐sided *t*‐test.

^§^ Mann‐Whitney U test.

PD, Parkinson's disease; UPDRS, Unified Parkinson's Disease Rating Scale; ESS, Epworth Sleepiness Scale; PSQI, The Pittsburgh Sleep Quality Index; RBDQ1, REM sleep behavior disorders single item questionnaire; FACIT‐F, Functional Assessment of Chronic Illness Therapy ‐ Fatigue scale; HADS, The Hospital Anxiety and Depression Scale; PKG, Parkinson's KinetiGraph.

Table 2 shows that motor fluctuators tended to have higher values on the study variables compared to the non‐fluctuators, except for KSS, sleepiness, BKS and PTI scores. There were stronger positive correlations between the individual average KSS and sleepiness values and other diary variables among fluctuators compared to non‐fluctuators (Table 2). The correlations between the within‐individual standard deviations (WISD) for the diary variables were also, overall, stronger among fluctuators than non‐fluctuators (Table 3). For both groups, the correlations between WISD for the diary variables and the PKG variables were generally weak and non‐significant. However, among fluctuators, higher FDS covaried with low WISD on sleepiness, while high DKS and FDS tended to coincide with a low WISD on anxiety ratings among non‐fluctuators (Table 3).

**TABLE 2:** *Descriptive statistics (upper part) and spearman correlations (bottom part) between diary and PKG variables among fluctuators and non‐fluctuators*

	1 ms	2 anx	3 mood	4 sleepiness	5 KSS	6 DKS_50	7 BKS_50	8 FDS	9 PTI
Fluctuators									
N	27	27	27	27	27	28	28	28	28
M	0.913	0.285	0.303	0.598	4.754	5.616	24.643	10.836	4.932
SD	0.355	0.409	0.375	0.436	0.961	7.627	7.589	4.753	4.108
Md	0.8	0	0.1	0.5	5	2.8	23.8	9.2	3.7
IQR	0.471	0.578	0.496	0.699	1.401	4.475	9.175	5.025	5.925
Min	0.3	0	0	0	2	0.2	12.4	4.2	0.1
Max	1.8	1.3	1.1	1.3	6.1	32.2	44.9	22.8	14.3
Non‐fluctuators									
N	23	23	23	23	23	24	24	24	24
M	0.621	0.146	0.105	0.588	4.445	2.067	29.994	7.454	7.158
SD	0.372	0.294	0.273	0.378	1.128	2.021	5.917	2.108	5.588
Md	0.5	0	0	0.6	4.4	1.4	30.7	7.3	5.2
IQR	0.654	0.111	0.065	0.693	1.511	1.950	3.725	2.800	4.950
Min	0	0	0	0	2.7	0.2	17	3.1	0.4
Max	1.1	1.2	1	1.3	7.4	7	41.3	13.2	23.1
Correlations for fluctuators (above diagonal) and non‐fluctuators (below diagonal)									
1	—	0.489**	0.662***	0.517**	0.451*	−0.203	0.429*	−0.217	0.198
2	0.301	—	0.774***	0.606***	0.534**	−0.152	0.248	−0.292	0.019
3	0.239	0.640**	—	0.505**	0.465*	−0.262	0.496**	−0.291	0.135
4	0.237	0.236	0.534**	—	0.688***	−0.281	0.311	−0.450*	0.148
5	0.159	0.035	0.089	0.510*	—	−0.257	0.357	−0.511**	−0.052
6	0.358	−0.619**	−0.368	−0.008	−0.062	—	−0.853***	0.841***	−0.703***
7	−0.022	0.398	0.228	0.085	0.091	−0.564**	—	−0.726***	0.630***
8	0.142	−0.631**	−0.373	−0.209	−0.234	0.632***	−0.349	—	−0.492*
9	−0.022	0.448*	0.359	0.432*	0.296	−0.531**	0.596**	−0.256	—

* *P* < .05.

** *P* < .01.

*** *P* < .001.

N, sample size; M, mean; SD, standard deviation; Md, median; IQR, interquartile range; Min, minimum; Max, maximum; ms, motor symptoms; anx, anxiety; KSS, Karolinska Sleepiness Scale; DKS 50, median dyskinesia score 9 am–6 pm; BKS 50, median bradykinesia score 9 am–6 pm; FDS, fluctuation score; PTI, percent time immobile.

**TABLE 3:** *Spearman's correlations of within‐individual standard deviations (wisd) of diary and PKG variables for fluctuators (above diagonal) and non‐fluctuators (below diagonal)*

	1	2	3	4	5	6	7	8	9
1 ms_wisd	—	0.154	0.398*	0.409*	0.285	−0.113	0.207	−0.196	−0.022
2 anx_wisd	0.247	—	0.797***	0.478*	0.257	−0.067	0.132	−0.235	0.001
3 mood_wisd	0.047	0.553**	—	0.473*	0.183	−0.135	0.354	−0.231	0.077
4 sleep_wisd	−0.001	−0.05	0.379	—	0.708***	−0.250	0.258	−0.504**	−0.047
5 KSS_wisd	0.125	−0.171	−0.038	0.580**	—	0.054	−0.050	−0.154	−0.305
6 DK_50	−0.186	−0.589**	−0.349	−0.101	−0.036	—	−0.855***	0.823***	−0.727***
7 BK_50	−0.111	0.382	0.182	−0.012	−0.275	−0.564**	—	−0.723***	0.644***
8 FDS	−0.158	−0.618**	−0.342	0.046	0.111	0.632***	−0.349	—	−0.514*
9 PTI	0.145	0.403	0.313	0.091	−0.163	−0.531**	0.596**	−0.256	—

* *P* < 0.05.

** *P* < 0.01.

*** *P* < 0.001.

wisd, within individual standard deviation; ms, motor symptoms; anx, anxiety; KSS, Karolinska Sleepiness Scale; DKS 50, median dyskinesia score 9 am–6 pm; BKS 50, median bradykinesia score 9 am–6 pm; FDS, fluctuation score; PTI, percent time immobile.

**FIG 1:**
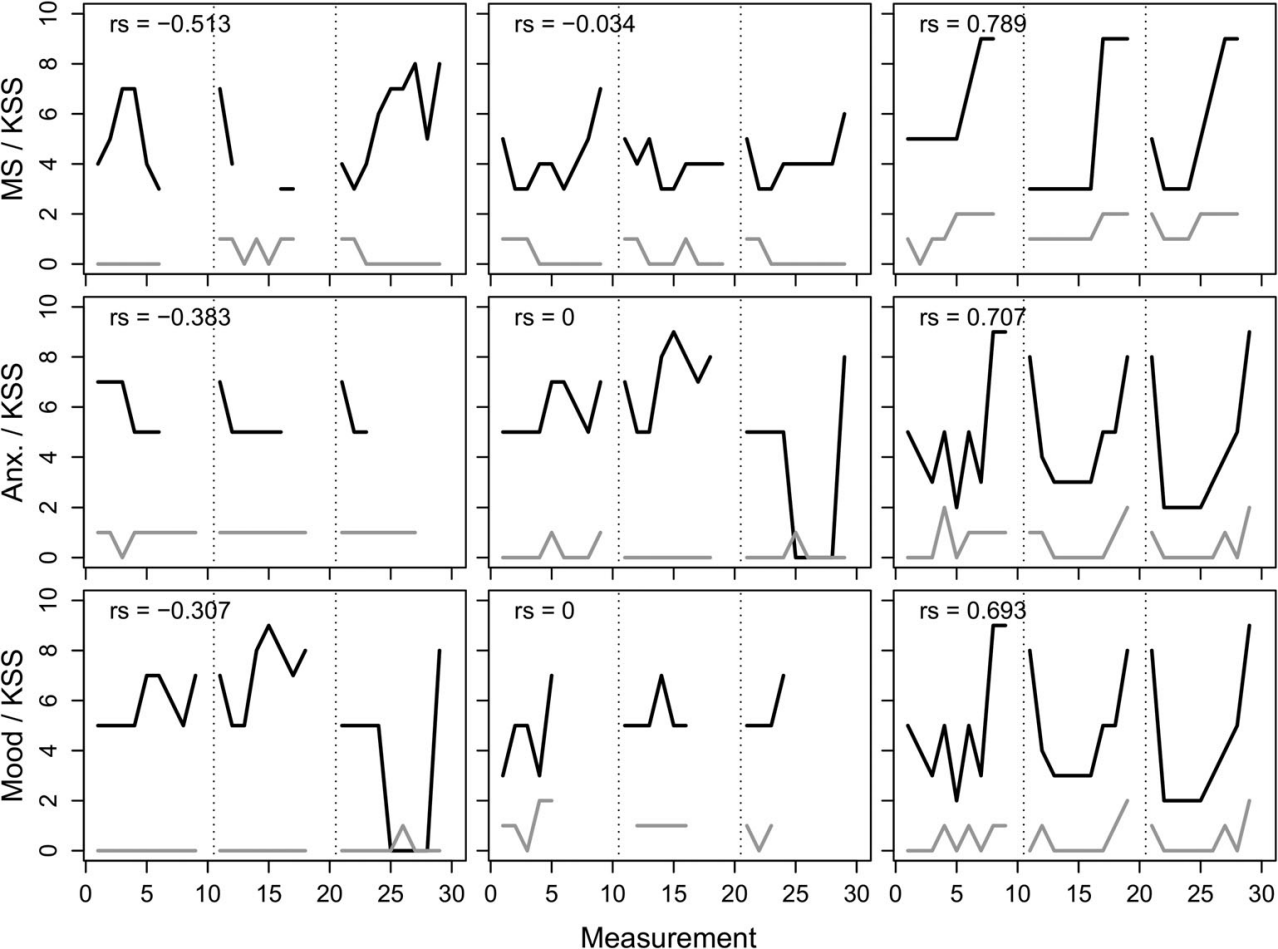
MS, motor symptoms; KSS, Karolinska sleepiness scale; Anx, anxiety. Trajectories of MS (gray lines, first row), anxiety (gray lines, second row), and mood (gray lines, third row) and KSS (black lines) over 30 possible measurements over 3 days (separated by dotted lines) for the participant with the strongest negative (first column) and positive (third column) as well as one participant with hardly any (second column) within‐individual correlation (Spearman's) between the two measures. N = 1 in each panel.

**FIG 2:**
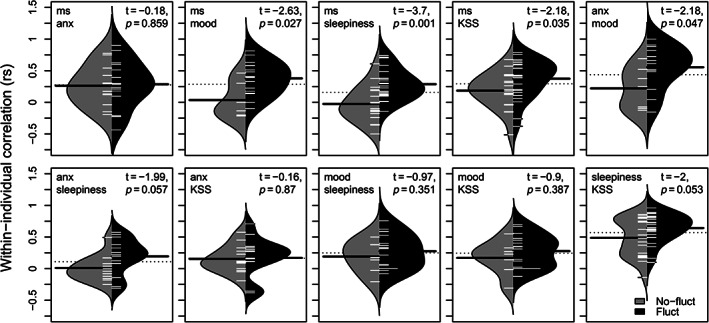
MS, motor symptoms; KSS, Karolinska sleepiness scale; Anx, anxiety. Density of observations of within‐individual correlations (WIRS) for fluctuators (black) and nonfluctuators (gray). In each panel, the dotted line indicates the grand mean of the WIRS, the two solid black lines indicate the mean WIRS in each subgroup, and the small white lines indicate individual values. The differences in WIRS between fluctuators and nonfluctuators have been analyzed with weighted (for within‐individual number of observation) two sample t‐tests (see the panels for t‐ and *P*‐values).

The within‐individual correlations (WIRS) tended to be positive, indicating that high ratings on one diary variable tended to coincide with high ratings on the other variables. However, for each pair of variables at least some participants exhibited negative WIRS as well (Fig. 1). WIRS tended to be stronger among fluctuators compared to non‐fluctuators (Fig. 2). For example, the WIRS between self‐rated motor symptoms and sleepiness was markedly stronger among fluctuators.The differences between average MMSE score for fluctuators and non‐fluctuators was small and not significant. Spearman's r was used for correlation analysis, and it means that eventual outliers did not have a strong impact on the results.

## Discussion

This study investigated the relationship between daytime sleepiness and other non‐motor and motor fluctuations using both a home diary and PKG registrations.

There is no consensus on how to classify PD patients as motor fluctuators. Different classifications have been used, which makes it difficult to compare results from different studies. For example, Ossig et al.^7^ used medical records in addition to the UPDRS part IV, whereas we defined participants as fluctuators or non‐fluctuators based on the UPDRS part IV, and interviews with them.

Using our definition, we found that fluctuations of daytime sleepiness, particularly as measured by KSS, correlated moderately with other fluctuations among participants classified as fluctuators, but weakly among non‐fluctuators. Overall, fluctuators showed stronger correlations between all diary variables (daytime sleepiness, anxiety, mood and motor symptoms) compared to non‐fluctuators. In agreement with previous work,^6^, ^7^ we found correlations between fluctuations in anxiety and mood both for motor fluctuators and non‐fluctuators. This probably reflects the high degree of co‐morbidity between anxiety and mood disorders.

Sleep disturbances and daytime sleepiness are influenced by treatment with dopaminergic medication.^4^ In the present study, fluctuators were treated with higher dosages of levodopa, but tended to have lower doses of dopamine agonists. The lower dosages of dopamine agonists among fluctuators probably relate to the fact that they had longer PD duration and more advanced PD. The treatment recommendation in Sweden according to national guidelines from the Swedish Movement Disorders Society is to reduce or even remove dopamine agonists when the disease progresses and especially when the people with PD receive advanced therapies.^27^ In our sample, nine fluctuators were having advanced treatment (seven treated with levodopa‐carbidopa intestinal gel and two on Deep Brain Stimulation, DBS) while only one non‐fluctuator was having treatment with DBS.

Interestingly, PKG data were generally weakly correlated with diary data. For example, FDS was the only PKG variable that correlated with daytime sleepiness WISD among fluctuators. It is interesting to compare our data with that of Kotschet and colleagues.^19^ They showed a correlation between PKG's PTI and ambulatory daytime polysomnography (PSG) and therefore suggested that PTI can be a useful surrogate measure of daytime sleepiness in PD. However, the result of Kotschet et al.^19^ is based on a low number of PSG records. Furthermore, it should be noted that it can be problematic to use PSG to detect daytime sleepiness because PSG only records periods of sleep. Daytime sleepiness is defined as a subjective experience of daytime sleepiness, a tendency to fall asleep, or nod off during the daytime,^10^, ^28^ which does not necessarily lead to falling asleep. Another study^29^ investigated whether PKG is a useful tool to detect disturbed night‐time sleep in people with PD with and without excessive daytime sleepiness (EDS). It was found that PKG measures were associated with insomnia, parasomnia and restless legs syndrome among people with PD and EDS, but not among those without EDS. While relevant for our study, it should be noted that EDS is more multifactorial than lack of night‐time sleep.^28^ Our present study focuses on daytime sleepiness from the people with PD perspective and not primarily on PKG as a tool to detect EDS or sleep disturbances in PD. Nevertheless, our diary data do not support that single usage of PKG is useful to detect daytime sleepiness, and further investigations and refinements on the topic are obviously warranted.

This study has some limitations. In particular, participants did not complete the home diary during the full PKG registration. The reason for this was our intention to minimize the risk of diary fatigue. For example, Hauser et al.^30^ found that missing and duplicate diary entries tend to occur after 3 days. An alternative to home diaries could have been electronic diaries, but their use is associated with additional challenges.^31^ Moreover, as indicated above, it is suboptimal to compare six‐day summaries of PKG data with diary data with multiple time points. That is, access to a higher time‐resolution of the PKG data to enable day‐by‐day, or even individual time point correlations would have been preferable. For example, it has been shown by Ossig et al.^32^ that shifting PKG data by 30 minutes resulted in a much better agreement with diary data on motor symptoms. Another limitation is that our cognitive assessment was relatively crude. We excluded people with PD with a dementia diagnosis, but it should be noted that moderate cognitive impairment can also have an impact on home diary completion.

In summary, motor fluctuators showed stronger correlations between all diary variables (daytime sleepiness, anxiety, mood and motor symptoms) compared to non‐fluctuators. Stronger positive within‐individual correlations were found among fluctuators in comparison to non‐fluctuators. Fluctuations of daytime sleepiness according to home diaries showed a significant correlation with other non‐motor symptoms, but correlated relatively poorly with accelerometer measures by single usage of PKG. Our observations provide novel evidence that daytime sleepiness is associated with other motor and non‐motor fluctuations in PD.

## Author Roles

(1) Research project: A. Conception, B. Organization, C. Execution; (2) Statistical Analysis: A. Design, B. Execution, C. Review and Critique; (3) Manuscript: A. Writing of the first draft, B. Review and Critique.

A.H.: 1A, 1B, 1C, 2A, 2B, 2C, 3A

P.H.: 1A, 1B, 2C, 3B

J.‐E.B.: 1B, 2C, 3B

S.E.P.: 1B, 2C, 3B

K.S.: 1B, 2A, 2B, 2C, 3B

S.F.: 1A, 1B, 2C, 3B

P.S.: 1B, 2C, 3A

## Disclosures

### Ethical Compliance Statement

This study was approved by the ethical review board at Karolinska Institutet, Sweden (Dnr. 2011/1866‐31/4 and 2015/761‐32). All participants provided written informed consent. We confirm that we have read the Journal's position on issues involved in ethical publication and affirm that this work is consistent with those guidelines.

### Funding Sources and Conflicts of Interest

This work was supported by The Swedish Parkinson Foundation (865/16). P.S. is a Wallenberg Clinical Scholar. The other authors declare that there are no conflicts of interest relevant for this work.

### Financial Disclosures for the Previous 12 Months

S.F. has received honoraria for lectures, educational activities and/or advisory boards during the preceding 12 months from Almiral, Biogen, Celgene, Merck, Novartis, Roche and Sanofi. The other authors declare that there are no additional disclosures to report.
